# Infant Cardiac CT Angiography with 64-Slice and 256-Slice CT: Comparison of Radiation Dose and Image Quality Using a Pediatric Phantom

**DOI:** 10.1371/journal.pone.0049609

**Published:** 2012-11-21

**Authors:** Yi-Wei Lee, Ching-Ching Yang, Greta S. P. Mok, Tung-Hsin Wu

**Affiliations:** 1 Department of Biomedical Imaging and Radiological Sciences, National Yang Ming University, Taipei, Taiwan; 2 Department of Radiology, Kaohsiung Chang Gung Memorial Hospital and Chang Gung University College of Medicine, Kaohsiung, Taiwan; 3 Department of Radiological Technology, Tzu Chi college of Technology, Hualien, Taiwan; 4 Department of Electrical and Electronics Engineering, Faculty of Science and Technology, University of Macau, Macau, China; The University of Tennessee Health Science Center, United States of America

## Abstract

**Background:**

The aims of this study were to investigate the image quality and radiation exposure of pediatric protocols for cardiac CT angiography (CTA) in infants under one year of age.

**Methodology/Principal Findings:**

Cardiac CTA examinations were performed using an anthropomorphic phantom representing a 1-year-old child scanned with non-electrocardiogram-gated (NG), retrospectively electrocardiogram-gated helical (RGH) and prospectively electrocardiogram-gated axial (PGA) techniques in 64-slice and 256-slice CT scanners. The thermoluminescent dosimeters (TLD) were used for direct organ dose measurement, while dose-length product and effective mAs were also used to estimate the patient dose. For image quality, noise and signal-to-noise-ratio (SNR) were assessed based on regions-of-interest drawn on the reconstructed CT images, and were compared with the proposed cardiac image quantum index (CIQI). Estimated dose results were in accordant to the measured doses. The NG scan showed the best image quality in terms of noise and SNR. The PGA scan had better image quality than the RGH scan with 83.70% dose reduction. Noise and SNR were also corresponded to the proposed CIQI.

**Conclusions/Significance:**

The PGA scan protocol was a good choice in balancing radiation exposure and image quality for infant cardiac CTA. We also suggested that the effective mAs and the CIQI were suitable in assessing the tradeoffs between radiation dose and image quality for cardiac CTA in infants. These results are useful for future implementation of dose reduction strategies in pediatric cardiac CTA protocols.

## Introduction

Congenital heart disease (CHD) is the most common birth abnormality and the leading cause of death for infants under one year of age. Although invasive catheter-directed cardiac angiography is currently the reference standard for the assessment of complex cardiovascular anomalies in infants, it has the potential to impart high doses of radiation and contrast medium to patients due to extended fluoroscopic and cine evaluation. Other disadvantages include patient discomfort, bad availability, costs of hospital stay and a small but non-negligible risk of complications such as stroke, vessel dissection and pseudoaneurysm formation. With advances in the multi-detector computed tomography (MDCT), the improvement in spatial and temporal resolution has allowed non-invasive evaluation of intrathoracic vessels, airways, cardiac anatomy and coronary arteries in infants with CHD [1]. Consequently, infant cardiac computed tomography angiography (CTA) has evolved to play a key role in evaluating complex CHD. However, radiation exposure with CTA has become an important issue due to the increased risk of cancer induction [2], [3], [4], [5]. In an average of all age groups, an estimated additional lifetime risk for developing cancer after 10 mSv exposure is approximately one in 2000 [6]. Although the link between medical radiation exposure and future cancer risk is still controversial, children are at a higher risk of suffering damage from radiation exposure than adults due to a longer life expectancy and higher radiation sensitivity. Hence, pediatric scanning protocols with parameters specifically designed for children are necessary.

Image degradation in CTA due to cardiac motion can be reduced by using electrocardiogram (ECG) gating, e.g. retrospectively ECG-gated helical (RGH) or prospectively ECG-gated axial (PGA) techniques [7], [8], [9]. Recently, it has been demonstrated that PGA-CTA performed with a body mass index (BMI) adapted protocol offers a significant reduction in radiation dose to <6 mSv [4], [5], [8], [9], [10]. However, there is not an established protocol for infant PGA-CTA. Pediatric cardiac CTA protocols are still under development even though the number of cardiac CTA examinations in infants is rapidly increasing. The recent introduction of the 256-slice CT scanner (Brilliance iCT; Philips Medical Systems, Eindhoven, Netherlands) with 270 ms gantry rotation and an 80 mm detector array has allowed a larger z-axial coverage and improved temporal resolution. In one pilot study of 256-slice PGA-CTA for infants with CHD reported by Huang *et al.*, diagnostic quality images could be achieved in all cases and their accuracy was up to 95.9% [11]. To the best of our knowledge, the performance of pediatric cardiac CTA protocols especially for infants has not yet been fully addressed. The purpose of this study was to investigate the image quality and radiation dose resulting from various pediatric cardiac CTA techniques, including non-electrocardiogram-gated (NG), RGH and PGA, performed with 64-slice and 256-slice CT for 1-year old infants. Furthermore, we also investigated the relationships among the protocol parameters, radiation dose and the image quality.

**Figure 1:**
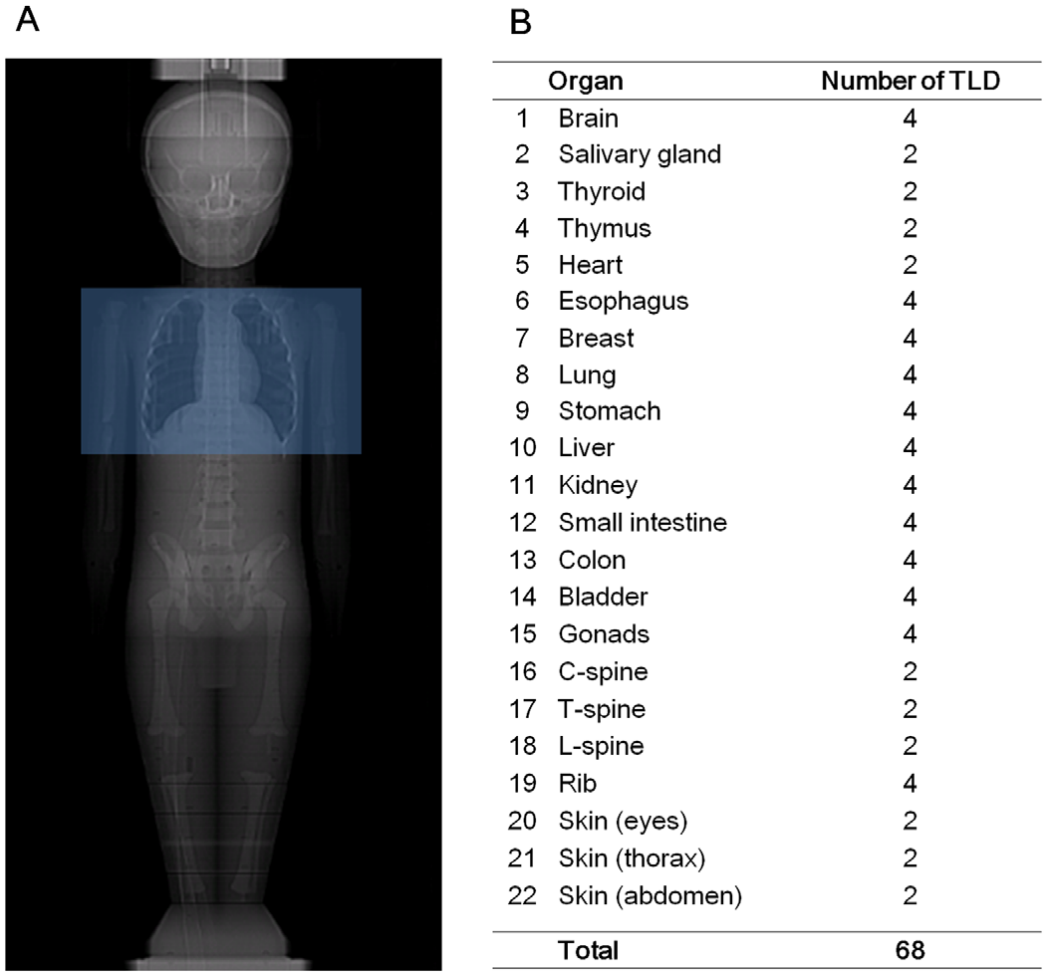
TLD locations in the anthropomorphic phantom. (A) The scan range included the entire lung from apex to base. (B) A total of 68 TLDs were placed at 22 phantom sites.

## Materials and Methods

### Pediatric Anthropomorphic Phantom

A tissue-equivalent anthropomorphic phantom representative of a 1-year-old child (ATOM, Model 704-D, CIRS, Norfolk, VA, USA) was used in this study. The pediatric phantom consists of 29 numbered transverse sections and 2 arm attachments. Each section is 25 mm thick and contains through-holes at various organ locations. The phantom contains 6 different tissue types: 1-year bone (physical density, ρ = 1.45 g/c.c.), soft tissue (ρ = 1.05 g/c.c.), spinal cord (ρ = 1.07 g/c.c.), spinal discs (ρ = 1.15 g/c.c.), lung (ρ = 0.20 g/c.c.) and brain (ρ = 1.07 g/c.c.).

### CT Data Acquisition

CTA scans were performed using a 64-slice CT (Brilliance CT, Philips Healthcare, Cleveland, OH, USA) with a collimation of 64×0.625 mm and a 256-slice CT (Brilliance iCT, Philips Healthcare, Cleveland, OH, USA) with a 2×128×0.625 mm collimation by means of a z-flying focal spot. The shortest gantry rotation time is 400 ms in the 64-slice CT and is improved to 270 ms in the 256-slice CT. The specific scanning parameters are summarized in Table 1, designed based on our preliminary clinical experience and previously published clinical information [1]. All scanning protocols used a fixed scan length of 137 mm with field-of-view of 150 mm to cover the entire lung of the pediatric anthropomorphic phantom [1] (Figure 1). The full scan reconstruction algorithm, in which data collected from 360° angular coverage were used to reconstruct an axial image, was employed for NG scans. Partial scan reconstruction algorithm, where data collected from either an angular coverage of 180° or 210° were used to reconstruction an axial image, was used for RGH and PGA scans to further improve temporal resolution. Patient cardiac simulator (Cardiac Trigger, Model: CTM300, IVY biological system, Inc.) was used to initiate electrocardiogram-gated scan modes, set at a rate of 120 beats per minute (bpm) representing a normal heart rate for 1-year old infants. In patients with heart rate >72 bpm, motion artifacts in the systolic phase was significantly lower than those in diastolic phase [12], so data acquired with RGH scans were reconstructed at 45% of R-R interval, whereas PGA scans were prospectively triggered at 45% of the R-R interval without padding as our clinical settings. The PGA scan mode was not available in our 64-slice CT scanner. Cardiac and respiratory motions were not modeled in our phantom.

**Table 1:** Imaging parameters for our pediatric phantom study.

Parameters	64-slice CT		256-slice CT		
	NG	RGH	NG	RGH	PGA
kV	80	80	80	80	80
mA	156	190	234	266	283
Tube rotation time (ms)	500	400	330	270	270
Tube shot time per rotation (ms)	500	400	330	270	210
Temporal resolution (ms)	500	200	330	135	180
Collimation (mm)	64×0.625	64×0.625	128×0.625	128×0.625	128×0.625
Pitch	0.67	0.2	0.67	0.18	1
Effective mAs	116.4	380	115.3	399	59.4
CTDI_vol_ (mGy)	2.2	7.5	2.4	8.2	1.4
Scan length (mm)	137	137	137	137	137
Slice thickness (mm)	0.67	0.67	0.67	0.67	0.8
FOV (mm)	150	150	150	150	150
Filter type	Standard(B)	XCC	Standard(B)	XCC	XCB
Reconstruction length (degree)	Full scan (360°)	Half scan (180°)	Full scan (360°)	Half scan (180°)	2/3 scan (240°)
CIQI (mAs⋅mm)	52.26	25.46	51.74	24.06	40.75

NG, non-electrocardiogram-gated; RGH, retrospectively electrocardiogram-gated helical; PGA, prospectively electrocardiogram-gated axial.

### Image Quality Analysis

**Figure 2:**
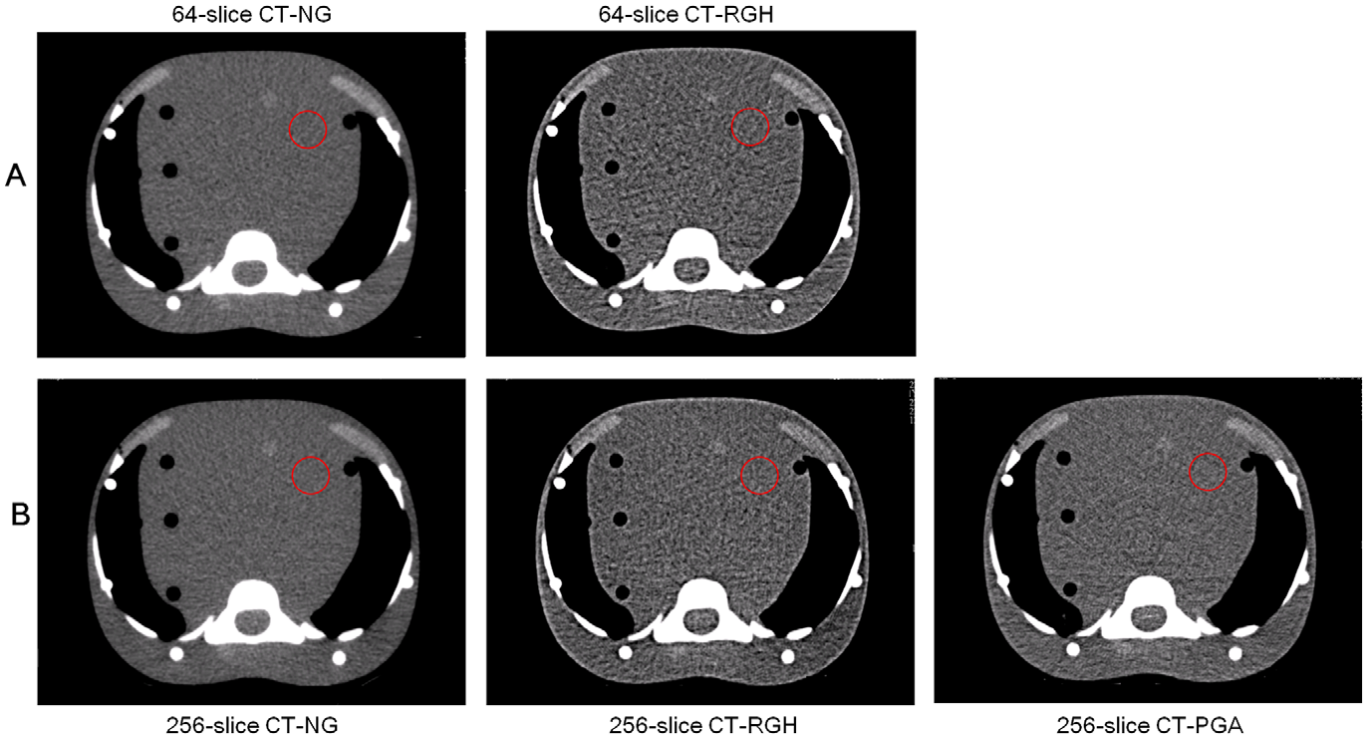
Axial CT slices of the pediatric anthropomorphic phantom at the level of the 8th thoracic vertebra for different image protocols: (A) 64-slice CT; (B) 256-slice CT. Red circle indicates a chosen ROI.

After end-systolic reconstruction, ten circular regions-of-interest (ROIs) with a diameter of 25 mm were placed in the left anterior mediastinum at the level of the 5^th^ to 10^th^ thoracic vertebrae to assess the image noise and signal-to-noise ratio (SNR) (Figure 2) for different image protocols. The noise defined in this study was the standard deviation (SD) of pixel intensities within ROIs. The SNR was determined by the ratio of the mean pixel values within ROI to its SD. To quantify the image noise due to photon counting statistics based on the acquisition parameters, a proposed cardiac image quantum index (CIQI) was defined as:
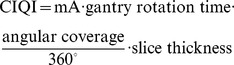


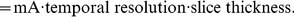
(1)


The effective tube current-time product (mAs), defined as the ratio between mAs and pitch, was also calculated to assess its correlation with image quality and radiation dose.

### Direct Thermoluminescent Dosimeters (TLD)-based Dose Measurement

The effective dose was estimated using direct organ dose measurement with LiF-TLD chips (TLD-100H, Bicron-Harshaw, Solon, OH, USA) in this study. Each point of dose measurement contained two to four TLDs (Figure 1). Ten TLDs were used to measure the dose to the red bone marrow located in the ribs, C-spine, T-spine, and L-spine. Bone marrow was used to estimate the doses received at the bone surface. For the assessment of skin dose, six TLDs were attached to the eyes, thorax and abdominal surface. Forty TLDs were placed in the regions of the brain, salivary gland, thyroid grand, esophagus, breasts, lungs, stomach, liver, colon, bladder and gonads. Twelve TLDs were placed in the thymus, heart, kidneys and small intestine to obtain an estimate of the absorbed dose to the remainder. According to guidelines published in the International Commission on Radiation Protection Publication (ICRP) Number 103 [13], the effective dose H_E_ is calculated as follows:
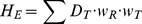
(2)


where 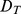 is the mean dose to the target organ, 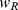 is the radiation weighting factor (for *x* ray, 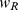 = 1), and 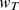 is the tissue weighing factor.

### Estimated Radiation Dose

Radiation dose estimates were determined using the volume CT dose index (CTDI_vol_) in Gy, as provided on the scanner console, and effective dose was expressed in mSv. The dose-length product (DLP), an indicator of the integrated radiation dose of an entire CT examination, is defined as the CTDI_vol_ multiplied by scan length. Calculation of estimated effective dose was obtained by multiplying the DLP by a conversion factor, k (0.026 mSv ⋅ mGy^−1^ ⋅ cm^−1^) as recommended by the American Association of Physicists in Medicine (AAPM) Report 96 [14].

## Results

**Figure 3:**
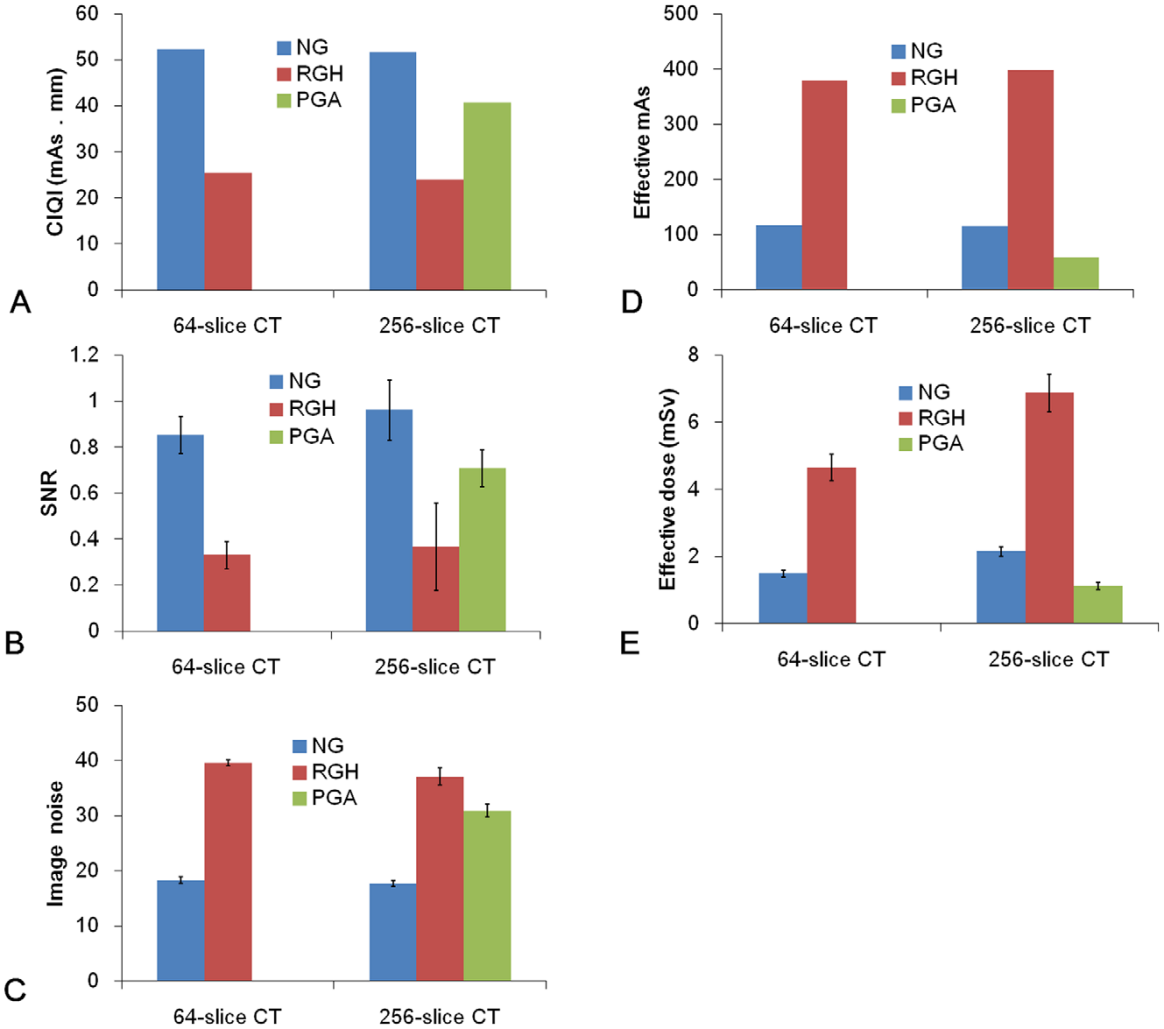
A comparison of (A) cardiac image quantum index (CIQI), (B) signal-to-noise ratio (SNR), (C) image noise, (D) effective mAs and (E) effective dose from TLD measurement for different imaging protocols.

**Figure 4:**
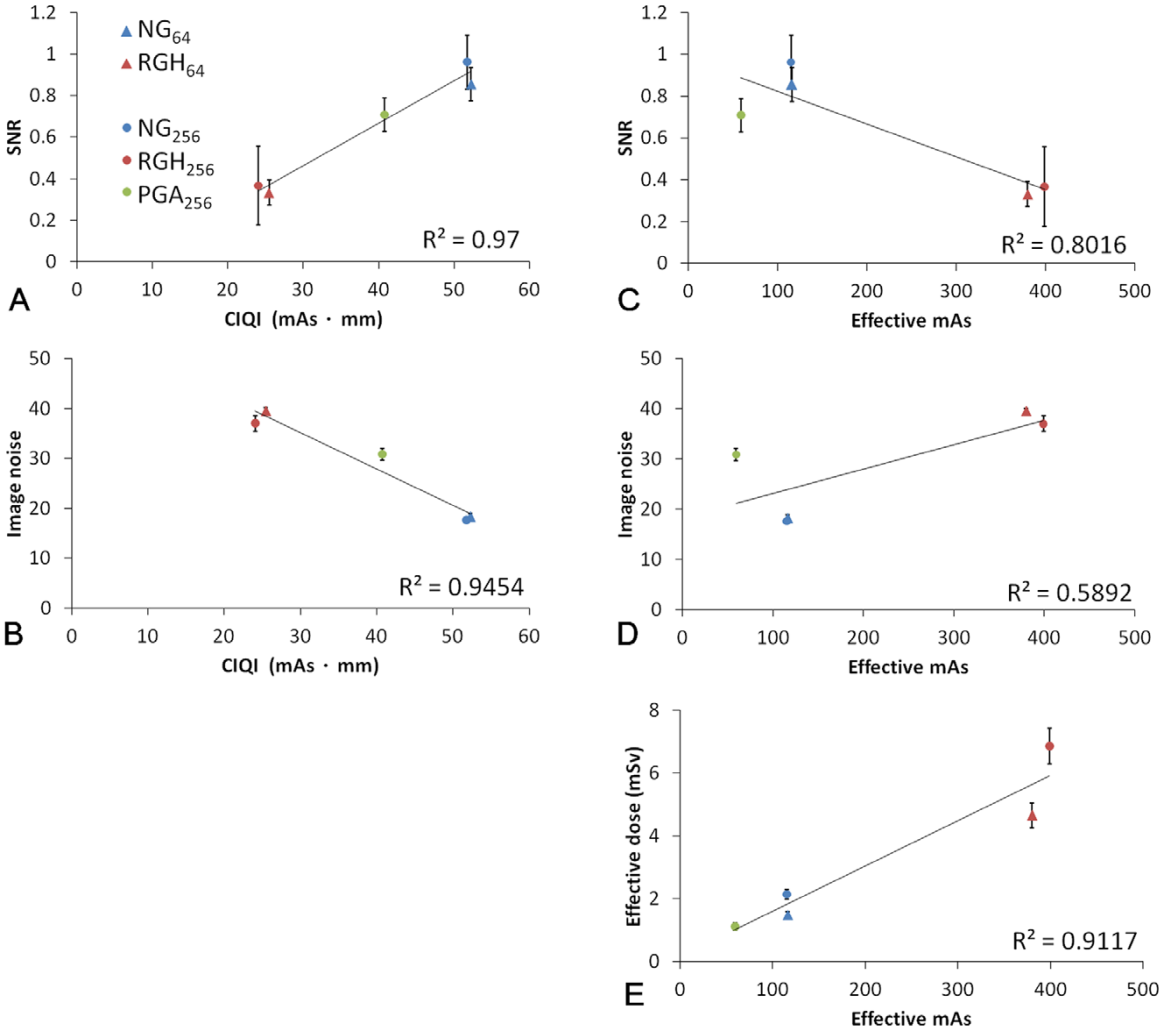
Linear regression analysis of (A) SNR versus CIQI, (B) image noise versus CIQI (C) SNR versus effective mAs, (D) image noise versus effective mAs, (E) effective dose from TLD measurement versus effective mAs. The correlations were calculated based on the whole 50 ROIs.

Figure 2 displays an axial slice of the pediatric anthropomorphic phantom at the level of the 8^th^ thoracic vertebra. Based on visual assessment, data acquired with NG scan on either scanner demonstrated slightly better image quality in terms of lower quantum noise. Among various protocols, the image noise due to photon counting statistics was most obvious for data acquired with RGH scans for both scanners (Figure 2), as confirmed by quantitative analysis of SNR and image noise (Figure 3). In 64-slice CT, image noise levels were 18.28±0.65, 39.57±0.57, and SNRs were 0.85±0.08, 0.33±0.06 for NG and RGH scans, respectively. In 256-slice CT, image noise levels were 17.65±0.53, 37.05±1.56, 30.90±1.19, and SNRs were 0.96±0.13, 0.37±0.19, 0.71±0.08 for NG, RGH and PGA scans, respectively. Images obtained using the NG protocol showed the best SNR and lowest image noise. The CIQIs of the five groups are summarized in Table 1. The NG protocols had highest CIQI scores no matter in 64-slice or 256-slice CTA scans and the RGH protocols had the lowest. The image noise and SNR were strongly associated with the CIQI. Our results showed that higher effective mAs did not assure better infant cardiac CTA image quality (Figure 4). There is not a significant difference in image quality indices between 64-slice and 256-slice CT for each acquisition mode in this study.

**Figure 5:**
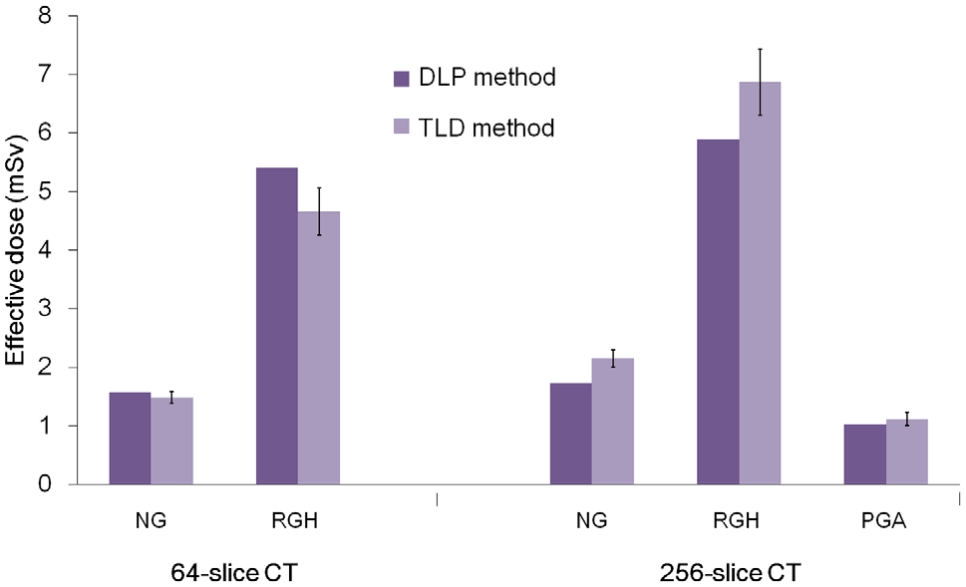
A comparison of the effective dose measured with DLP method and TLD method.

Based on the TLD results and ICRP 103 tissue weighted factors, effective doses for five different scan protocols were calculated and shown in Table 2. In 64-slice CT, effective doses were 1.49±0.10 and 4.66±0.40 mSv for NG and RGH scans, respectively. In 256-slice CT, effective doses were 2.15±0.15, 6.87±0.56 and 1.12±0.11 mSv for NG, RGH and PGA scans, respectively. The PGA scan had better image quality than the RGH scan with 83.70% dose reduction in gated CTA protocols. The highest organ doses from all protocols were to the lungs and the breasts. The breast dose and lung dose for the PGA protocol were 2.04 mGy and 1.80 mGy respectively, which were lower than those from the NG and RGH protocols regardless of 64-slice or 256-slice CTA scan. Figure 4 show a comparison of effective mAs and measured effective dose for both scanners. It indicates that the effective mAs was positively related to the patient radiation dose. The effective doses calculated from the DLP method were higher than those obtained from TLD measurement in 64-slice CTA scans, whereas the difference ranged from 5.7 to 13.7%. On the contrary, the dose estimates from the DLP method were 8.7 to 16.4% lower than those obtained from TLD measurement in 256-slice CTA scans (Figure 5).

**Table 2:** Organ dose and effective dose values obtained from TLD measurements for different acquisition protocols.

	64-slice CT		256-slice CT		
Organ dose (mGy)	NG	RGH	NG	RGH	PGA
Brain (0.01)	0.10±0.01	0.34±0.06	0.27±0.01	0.63±0.04	0.10±0.01
Salivary gland (0.01)	2.36±0.02	7.76±0.04	4.00±0.05	13.51±0.95	1.89±0.07
Thyroid (0.04)	2.54±0.02	7.76±0.04	4.00±0.05	13.50±0.95	2.03±0.07
Thymus (0.04)	2.66±0.10	7.69±0.31	3.22±0.02	12.02±0.00	2.21±0.13
Esophagus (0.04)	2.18±0.09	7.21±0.52	3.13±0.09	11.29±1.72	1.96±0.40
Breast (0.12)	2.34±0.05	7.35±0.18	3.60±0.23	10.74±0.03	2.04±0.06
Lung (0.12)	2.26±0.09	7.50±0.93	3.16±0.27	10.66±0.62	1.80±0.31
Stomach (0.12)	2.15±0.36	6.47±0.91	2.60±0.16	9.95±0.96	1.39±0.07
Liver (0.04)	1.37±0.12	4.28±0.40	2.47±0.33	5.60±1.75	0.52±0.03
Kidney (0.04)	1.10±0.04	3.37±0.57	2.18±0.28	3.91±1.31	0.44±0.04
Small intestine (0.04)	0.24±0.01	0.71±0.04	0.42±0.09	0.71±0.08	0.14±0.03
Colon (0.12)	0.13±0.02	0.35±0.03	0.24±0.04	0.40±0.04	0.09±0.03
Bladder (0.04)	0.14±0.09	0.20±0.01	0.09±0.01	0.23±0.00	0.07±0.00
Gonads (0.08)	0.04±0.00	0.09±0.00	0.03±0.00	0.08±0.00	0.03±0.02
Bone marrow (0.12)	1.67±0.14	5.27±0.49	2.40±0.15	7.50±0.78	1.17±0.15
Skin (0.01)	1.20±0.37	3.49±1.16	1.89±0.57	4.22±1.85	0.90±0.54
Bone surface (0.01)	1.67±0.14	5.27±0.49	2.40±0.15	7.50±0.78	1.17±0.15
Effective dose (mSv)	1.49±0.10	4.66±0.40	2.15±0.15	6.87±0.56	1.12±0.11

Data are presented as mean ± standard deviation.

A standard deviation value of 0.00 represents that it is less than 0.004.

The number in parentheses is the tissue weighting factor 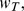 defined in ICRP 103.

## Discussion

Although technologic innovation often heralds new or improved diagnostic benefits, it is prudent to approach these benefits with an understanding of additional costs and risks. For CT, the main concern would be the radiation dose. As CTA and gated-cardiac CTA become increasingly popular for the cardiovascular evaluation in the pediatric population [1], [15], knowledge and understanding of the radiation dose are imperative. This is in part due to both increased radiosensitivity in the pediatric population and their longer lifetime for radiation-induced stochastic effects [16], [17], [18], [19]. Trade-off in radiation exposure and image quality for comprehensive assessment of the whole heart remains a difficult decision. Therefore, establishing references of MDCT radiation doses for children is necessary for both clinical management and research.

Our results showed that the doses from RGH cardiac CT are the highest to a 1-year-old and even exceed 6 mSv for 256-slice CT. These doses for infants are well beyond those used for routine chest CT in adults (5.4 mSv) or children (<5 mSv) [20], [21]. In addition, the dose is also higher than median effective radiation doses (4.6 mSv) for pediatric cardiac catheterizations and is about between the 50^th^ and 75^th^ percentile for infants [22], [23]. On the other hand, the doses of other protocols are low in our study, especially with PGA technique on 256-slice CT (1.12 mSv). Generally, radiation dose in MDCT is considered as being proportional to effective mAs, and our study showed similar results. In a helical CT scan, a pitch less than 1 implies that x-ray beam partially overlaps in the axial direction, so the decrease of pitch causes more x-ray photons to penetrate the patient at a specific axial location and thus increases patient radiation dose. Due to the x-ray beam overlaps, redundant data at a specific location can be acquired by different detector rows of a MDCT. In non-cardiac CT examination, combining redundant data for the same axial image would reduce image noise. However, the redundant data are acquired in consecutive heart cycles for cardiac CT, so data averaging results in degraded temporal resolution. Instead, data acquired from different heart cycles are used to reconstruct images at different axial positions in cardiac CT. Therefore; the number of photons contributing to the cardiac reconstruction depends only on the mAs, not the pitch. Our results also showed that higher effective mAs did not associate with improved image quality of infant cardiac CTA (Figure 4). In order to achieve full anatomical coverage in axial direction, a small pitch (approximately 0.2 to 0.3) needs to be used in RGH CT scan, but it also results in a relatively higher patient radiation dose.

There are several dose reduction strategies for pediatric patients, including tube current modulation, a child-size bowtie filter and scanning FOV, a weight or size-based technique chart that determines the appropriate kV and mA for children. However, there is a tradeoff between radiation dose and image quality. As effective mAs can be used to predict the radiation dose but it does not associate with image quality in cardiac CT, we have defined the CIQI to assess image quality of cardiac imaging before actual acquisition. In CT, image noise and SNR are influenced by many technical parameters, such as mA, scan time, kV, pitch, voxel size, patient size and reconstruction algorithm. As described above, the image quality in cardiac CT is independent of pitch, so pitch is not included in the definition of CIQI. One of the major components that would determine image quality in CT is the number of detected x-ray photons. Increasing mA, gantry rotation time and slice thickness can improve image quality by reducing quantum noise, so these factors were taken into considerations when defining the CIQI. Scanning techniques and reconstruction algorithms also play important roles in influencing image quality. In order to achieve high temporal resolution, partial scan reconstruction algorithm was employed to acquire the minimum amount of scan data needed for reconstruction. When the angular coverage used in partial scan is reduced from 360° to 180° plus fan angle, the data used to reconstruct an axial image are collected with an acquisition time shorter than the gantry rotation time, hence improving temporal resolution. On the other hand, due to the shorter partial-scan acquisition time, the number of x-ray photons reaching the detector is correspondingly reduced. Therefore, the ratio between angular coverage and 360°, which is derived from the ratio between partial-scan acquisition time and gantry rotation time, was included in the definition of CIQI. As shown in Figure 3 & 4, images with higher CIQI demonstrated lower noise and higher SNR, and vice versa. Therefore, the proposed CIQI should be suitable for assessing the impact of parameter modification on image quality for cardiac CT examination. Overall, the highest radiation dose was observed in data acquired with RGH, followed by NG and PGA. This finding suggested that, among various scan parameters, pitch is the most dominant factor affecting radiation dose. On the other hand, the best SNR was shown in data acquired with NG, followed by PGA and RGH. This result indicated that the angular coverage used to collect data for data reconstruction is the most dominant factor in image quality.

Besides a NG cardiac examination, if an additional ECG-gated cardiac examination is also needed for pediatric patient with suspected CHD, patient-specific modification on scanning parameters can be made to reduce radiation dose. Generally, reduced mAs and increased pitch are the most commonly used modification. Prior to performing the ECG-gated cardiac examination, radiological technologists and radiologists may be able to determine the most appropriate dose reduction strategy by investigating the impact of protocol modification on patient radiation dose and image quality by using the effective mAs and the CIQI, respectively. Based on our phantom study, the PGA scan protocol on 256-slice CT was the preferable choice in balancing radiation exposure and image quality in infant cardiac CTA. Hopefully, a cardiac CT examination that adheres to the ALARA (as low as reasonably achievable) principle could also maintain image quality for diagnostic use.

Balancing between reduction of radiation exposure and maintenance of diagnostic accuracy of infant CTA remains a difficult decision. The specific clinical information and main diagnostic problem are needed to determine the appropriate CTA protocol to provide a comprehensive evaluation of the respiratory and cardiovascular anatomy, including the coronary arteries. Even with a NG scan protocol to diagnose neonatal congenital heart disease, it still achieved very high accuracy and proximal coronary visualization was possible in >80% of pediatric cases using 16-slice CT [1], [24]. The RGH scans have been shown to significantly improve coronary artery visualization in infants and young children with CHD (LCA, >90%; RCA, >80%) [25]. Huang et al. [11] demonstrated that PGA-CTA for infants with CHD allowed detection of 98.8% of proximal coronary artery segments and 80% of the distal coronary artery segments. In addition, diagnostic quality images could be achieved in all cases and their accuracy was up to 95.9%. Accordantly, our study showed that the PGA scan protocol showed superior performance for cardiac imaging in infants on 256-slice CT, with significant dose reduction as compared to RGH scan as X-ray tubes are turned on only during a small predefined cardiac phase.

There are benefits of CT versus conventional catheterization in infants. Cardiac catheterization is invasive and associated with complications such as femoral artery occlusion, dissection, pseudoaneurysm formation, retroperitoneal hemorrhage, and serious sequelae such as limb growth discrepancy in infants and young children [21], [22], [23], [26], [27]. Cardiac catheterization may also require general anesthesia or sedation. Cardiac CTA does not require the same frequency of sedation as conventional angiography does and requires only an adequate venous access.

Cardiovascular magnetic resonance (CMR) and MDCT can complement the diagnostic information obtained by echocardiography and invasive cardiac catheterization. CMR imaging allows flow measurement, anatomic and functional evaluation without ionizing radiation. However, it is time-consuming and an overview of the relations between adjacent structures requires good and extended breath-holding for a series of studies in different planes. Thus, CMR for small infants with CHD is challenging and usually needs sedation on the patients. Its voxel size is also larger than that of the MDCT. Therefore, CTA is the modality of choice for patients who are unable to undergo CMR or are not suitable for general anesthesia [28], [29], [30].

We used an estimated conversion factor of 0.026 mSv ⋅ mGy^−1^ ⋅ cm^−1^ from AAPM Report 96 [14] for estimating effective dose in DLP method. The results were accordant with the phantom-base dose measurement. This conversion factor was slightly conservative when used in 256-slice infant cardiac CTA. Although the 256-slice CT can automatically choose an optimal detector collimation to minimize the number of steps and reduce the amount of z-overscan, the larger z-axis detector array and cone beam angle may lead to more scatter radiation and more additional irradiation outside the imaged volume [31]. This might explain why the measured doses were higher in 256-slice CT than that in 64-slice CT for different protocols in our study.

Our investigation has following limitations. First, we only evaluated image quality by an anthropomorphic phantom but not on real patients. The influence of cardiac and respiratory motion or stair-step artifacts on the image quality was not taken into consideration. Second, the examinations were all performed on CT scanners from a single vendor with selected protocols based on our clinical settings. The result may differ with scanners from a different vendor or dual-source CT. The data for PGA in 64-slice CT was not available for comparison. Moreover, the data provided here are really just a starting point prior to clinical studies and to keep in mind that the SNR and CNR may be very low, but that this may not have an impact of patient care, which is still clinically acceptable.

In conclusion, technical advances in cardiac imaging with cardiac CTA are rapidly evolving. Pediatric patients are at the greatest risk of developing cancer from radiation exposure, so dose reduction in pediatric cardiac CT examination is of great importance. While patient radiation dose can be reduced significantly by using various dose reduction strategies, it is also important to maintain diagnostic image quality. We have investigated the impact of scanning protocols for pediatric cardiac CT examination on patient radiation dose and image quality, in terms of noise and SNR. It was observed that radiation dose in cardiac MDCT scanning is proportional to the effective mAs. In addition, the performance of noise and SNR can be predicted by the CIQI. Based on these findings and the actual measurements, the PGA scan protocol on 256-slice CT was the preferable choice in balancing radiation exposure and image quality in this infant cardiac CTA phantom study. Besides, the effective mAs and the CIQI should be suitable in assessing the tradeoffs between radiation dose and image quality for cardiac CT. Optimal scanning parameters, dose reduction and assessment of the diagnostic accuracy of this technique are needed before its widespread use in the pediatric population. Further investigations to address the limitations of this study are warranted.
